# Meaningful syntactic structure in songbird vocalizations?

**DOI:** 10.1371/journal.pbio.2005157

**Published:** 2018-06-04

**Authors:** Johan J. Bolhuis, Gabriel J. L. Beckers, Marinus A. C. Huybregts, Robert C. Berwick, Martin B. H. Everaert

**Affiliations:** 1 Cognitive Neurobiology and Helmholtz Institute, Department of Psychology, Utrecht University, Utrecht, The Netherlands; 2 Department of Zoology and St. Catharine’s College, University of Cambridge, Cambridge, United Kingdom; 3 Utrecht Institute of Linguistics, Utrecht University, Utrecht, The Netherlands; 4 Department of Electrical Engineering and Computer Science, Massachusetts Institute of Technology, Cambridge, Massachusetts, United States of America

## Abstract

The faculty of language is thought to be uniquely human. Recently, it has been claimed that songbirds are able to associate meaning with sound, comparable to the way that humans do. In human language, the meaning of expressions (semantics) is dependent on a mind-internal hierarchical structure (syntax). Meaning is associated with structure through the principle of compositionality, whereby the meaning of a complex expression is a function of the meaning of its constituent parts and the mode of composition. We argue that while recent experimental findings on songbird call sequences offer exciting novel insights into animal communication, despite claims to the contrary, they are quite unlike what we find in human language. There are indeed remarkable behavioral and neural parallels in auditory-vocal imitation learning between songbirds and human infants that are absent in our closest evolutionary relatives, the great apes. But so far, there is no convincing evidence of syntax-determined meaning in nonhuman animals.

Birdsong has become a prominent animal model system for investigating the evolution and mechanisms of human speech and language. This is mainly due to some remarkable behavioral and neural parallels between the acquisition of spoken language (speech) in human infants and song learning in juvenile songbirds [1,2] that are absent in our closest relatives, the great apes. Three recent studies [3–5] claim to have found clear evidence that songbirds—the Japanese tit (*Parus minor*) and the southern pied babbler (*Turdoides bicolor*)*—*possess a humanlike ability to syntactically combine vocalizations to create more complex meanings. In other words, these animals are suggested to possess a sound-structure-meaning mapping similar to human language competence. Such results are extremely interesting, illustrating how comparative biological research might shed light on what is uniquely human to language, as well as gaining insight on the evolution of speech and language [6]. However, these results also demonstrate the difficulties involved in investigating “syntax” in nonhuman animals. We argue that these experiments provide novel insights into the use of vocal sequences in songbird communication but that evidence for sequencing these vocalizations according to principles underlying human (natural) language syntax (Box 1) remains lacking.

**Box 1: Language as a computational cognitive mechanism.**

Within the generative linguistic research tradition, language as a human cognitive trait is distinguished from language in a specific manifestation, say English. Language is taken as a computational cognitive mechanism, internal to the mind, and should not be conflated with a language having a possible function (e.g., communication) or a possible mode of externalization (e.g., speech). Language’s basic property is that it generates an unbounded array of hierarchically structured expressions.

Hierarchical structure in natural language follows from the operation Merge [9], a (dyadic) operation that takes two syntactic objects (call them X and Y) and constructs from them a single new syntactic object (call it Z). X and Y can be building blocks that are drawn from the lexicon or previously constructed objects. Put simply, Merge (X,Y) just forms the set {X, Y}, containing X and Y. Neither X nor Y is modified in the course of the operation Merge. E.g., *Eric can speak Russian* has hierarchical structure that derives from reapplication of Merge as in:

M(*Eric*, M(*can*, M(*speak*, *Russian*))) =

M(*Eric*, M(*can*, {*speak*, *Russian*})) =

M(*Eric*, {*can*, {*speak*, *Russian*}}) =

{*Eric*, {*can*, {*speak*, Russian}}}

Such hierarchical structures are interpreted at two interfaces: a conceptual-intentional interface, essentially having to do with meaning or “thought,” and a sensorimotor interface, concerned with the externalization of language as speech, sign, or some other modality. There is an asymmetry in “mapping” syntactic structure to the two interfaces. Language can, therefore, be taken as primarily meaning that may or may not be linked to sound or sign as an ancillary property. Mapping to the conceptual-intentional interface is direct, in the sense that the syntactic system structures thought. In a relevant sense then, the basic property of the language faculty generates and expresses thought. In contrast, mapping to the sensorimotor interface is complex, as internal hierarchical structure has to be “flattened” to linear strings that can be expressed as speech or sign. The linear nature of externalized language is a result of the physical constraints imposed by the sensorimotor systems. Thus, what reaches the senses is not what reaches the mind, and the linear structure of externalized language has a very limited role to play in semantics, if at all.

## Claims for compositionality in songbird vocalization

In their two studies, Suzuki and colleagues [3,5] build on earlier work [7] describing the “chicka” call system of the Japanese tit. These birds have a repertoire of 11 notes, which they use to produce chicka alarm calls in predator contexts. A call can consist of one note or a combination of notes. The particular combinations that are produced are highly variable. In two contexts alone, 175 different combinations occurred, and the usage of specific combinations can vary depending on the predator. In this sense, it is clear that the Japanese tit vocal communication system constitutes a potentially very interesting and relevant paradigm for the comparative study of language evolution [1,8].

In the first of their two studies, Suzuki and colleagues [3] showed that Japanese tits responded differentially to at least some of their naturally occurring calls: an “alert” call consisting of the notes ABC elicited “scanning” behavior, while a “recruitment” call consisting of just the D note elicited “approaching the caller.” When the birds heard the ABC call notes combined with the D call note as the compound ABC-D, also a natural vocalization, they showed both scanning and approach behaviors. However, there was little or no response when call order was artificially reversed to D-ABC. From this, the authors concluded that the combination ABC-D must have a compound meaning, because if ABC-D were interpreted by the birds as two separate calls in close proximity, then the reversal D-ABC should also elicit a corresponding response.

In a subsequent study [5], the authors added an interesting twist to their experimental design. They made use of the fact that Japanese tits often forage in mixed flocks with willow tits (*Poecile montanus*). In the wild, the willow tit “recruitment” tää call elicits approach, not only in willow tits but also in Japanese tits. When the authors played an artificial compound stimulus “ABC-tää” to the Japanese tits, the birds responded to this novel compound in a similar way (i.e., with both scanning and approach behaviors) as they did to ABC-D (in the authors’ previous study). When, in a control condition, the Japanese tits were exposed to the artificial compound stimulus—“tää-ABC”—the birds did not respond, similar to the D-ABC compound stimulus in Suzuki and colleagues’ previous study [3]. Willow tits also have an A—“zi.” When Japanese tits were exposed to the artificial compound call —“ABC-zi” or “zi-ABC”—the birds did not respond. Thus, it is unlikely that the ABC call acted as a kind of “priming” stimulus that would lead to responding to any compound call beginning with ABC. In addition, the birds also did not respond to an artificially shortened tää call (shortened by 50% to match the duration of D calls), suggesting that acoustic similarity could not account for the results.

These experiments with heterospecific calls are very interesting because they tell us more about the nature of the Japanese tit’s vocal communication system. As the authors suggest, the findings render it unlikely that the birds perceive the ABC-D compound as one stimulus with a compound meaning. Rather, it would appear that the animals interpret call sequences “by assessing and combining the meanings of individual call units (alert + approach)” [5].

Shortly after the first study by Suzuki and colleagues [3], Engesser and colleagues [4] published results describing a study with a similar experimental design using pied babblers. These birds have a vocal repertoire of approximately 17 different calls. Similar to Japanese tits [3], in natural contexts, the babblers could produce alert calls (in response to “suddenly appearing non-dangerous subjects”), recruitment calls (resulting in approaching the caller), and a combination of these two calls, which the authors term a “mobbing sequence” (the authors call this “M,” but for reasons of clarity, we will term this “AR”). In the experiment, the birds were presented with playbacks of these three calls, as well as an artificially constructed compound call CR consisting of a foraging “chuck” call C followed by the recruitment call R, as a control condition. The subjects’ response to the different calls was investigated using a number of behavioral measures. Overall, there was very little responding to alert call A and significantly greater responding to recruitment call R. There was a considerably greater response to the compound call AR (compared to the response to recruitment call R) but virtually no response to the artificial novel compound call CR. Thus, the basic experimental design of this study is similar to that of Suzuki and colleagues’ studies [3,5], but the result is different. While, in Suzuki and colleagues’ studies, the response to the compound stimulus was a combination of the responses to the two elements, in Engesser and colleagues’ study [4], the response to the compound is quantitatively different from the response to each of the individual elements.

Taken together, all three studies [3–5] find differential responsiveness to a compound (alert-recruitment) call compared to the individual elements of that call. In their second study, Suzuki and colleagues [5] found that replacing the conspecific recruitment call with a heterospecific recruitment call has a similar effect, suggesting that it is not acoustic similarity but similarity of meaning that causes the birds to respond to this novel combination. In the three studies [3–5], reversing the order of the compound (alert-recruitment) call [3,5] or replacing the alert call in the compound with a foraging call [4] did not lead to significant responding. In all three studies, it is claimed that the results invite the conclusion that the birds exhibit a form of compositionality, by which the meaning of call combinations is different from the meaning of the elements that make up the compound. Suzuki and colleagues [3,5] take this inference a step further, suggesting that the sequential order in which the calls are placed in the compound stimulus determines the meaning of this stimulus. In the remainder of this essay, we critically discuss what the authors of these three studies [3–5] claim: whether such sound combinations are really comparable to what happens in human language, both from a structural perspective (syntax) and from a meaning perspective (semantics).

## Syntax/structure and semantics/meaning

First, we summarize the linguistic assumptions underlying the authors’ [3–5] interpretation regarding the essential nature of human natural language syntactic structure. Engesser and colleagues [4] are quite clear on how they interpret their findings. They concentrate on how the syntax of human language (i.e., how an utterance is structured) contributes to semantics (i.e., what that utterance means). While humans combine word-like atomic elements—the building blocks of language—into phrases or sentences, pied babblers are capable of combining vocalizations into larger sequences. To be more precise, the authors talk about “flexible and productive concatenation of meaningful signals.” These combinatorial capabilities are taken as an “early form of human syntactic communication” and, for them, show “evidence for rudimentary compositionality.” Suzuki and colleagues [3] also take this route, compositionality combined with productivity: “its combinatorial power, which allows us to generate innumerable expressions from a finite number of vocal elements and meanings.” For them, therefore, the possibility to express “limitless meanings from a finite set of words based on combinatorial rules” is central to human language and crucial for their interpretation of their experiments.

All three papers introduce—correctly, in our view [9]—a special connection between syntax (how an expression is built) and semantics (what that expression means), and they take these properties of natural language as central in their analysis of birdsong. Note, however, that in their first paper, Suzuki and colleagues [3] observe that syntax, and thus the semantics attached to it, is unbounded, while it is unclear whether Engesser and colleagues [4] take that step. However, we will show that, in both cases, the concept of “semantically compositional syntax” is quite distinct from standard use of the term in linguistics.

## The nature of compositionality

**Fig 1:**
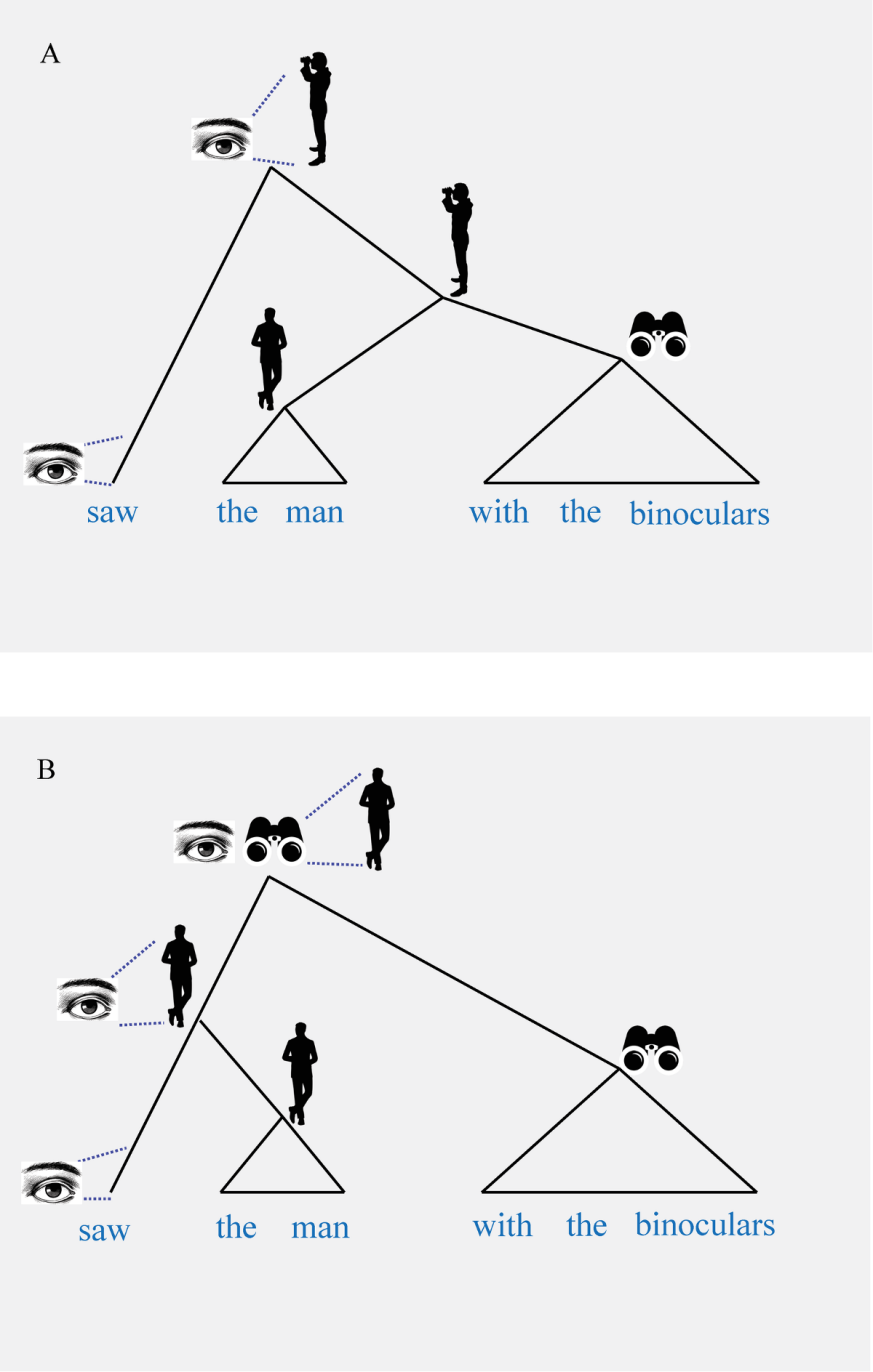
Meaning depends on syntactic structure. In Fig 1A, the phrase consists of the verb *saw* and the object of the verb *the man with the binoculars*, itself consisting of the noun phrase the man and a modifying prepositional complement *with the binoculars*. First, the modifying complement and the noun phrase are combined in what will be the object of the verb. The phrase in Fig 1B consists of a verb (*saw*), the object of the verb (*the man*), and an adjunct (*with the binoculars*), the means by which the man is seen. First, the object is combined with verb, and then this result is combined with the adjunct.

The notion of “compositionality” means that “the meaning of an expression is a function of the meanings of its parts and of the way they are syntactically combined” [10], as illustrated in Fig 1.

The noun *compositionality* is derived from the verb *to compose*, but the meaning of the former adds something to the meaning of the latter. I.e., the compositional meaning of, e.g., *very old red houses* is more than simply the summation of the meanings of *very*, *old*, *red*, and *houses* that the phrase is “composed of.” What is essential in this case is that both *old* and *red* modify *houses* but that *very* only modifies *old* and not *red*, a consequence of how the noun phrase is structured. On the other hand, if we say that *this soup is composed of pink-rimmed fish cake*, *daikon*, *carrot*, *and shiitake mushrooms*, we do not mean that the soup is made “compositionally” but rather that the soup is a potpourri.

The distinction between compositional semantics of human language and the combinatorial enrichments of vocalizations of songbirds and the alarm calls of West African Campbell monkeys [11] is fundamental and, in an interesting way, comparable to the principled distinction in chemistry between compounds (e.g., the liquid H_2_O resulting from a chemical reaction between two gases, oxygen and hydrogen) and mixtures (e.g., saline water solutions or milk emulsions).

Mixtures have no analogue in what has been called “lexical syntax” [12], such as (linguistic) compounds. Observe the combinations of the words *smart*, *phone*, and *company* in *smart phone company*. The possible meanings of this compound are not associative meanings such as “phones for smart companies,” “a smart company with phones,” “company phones that are smart,” etc. There are, necessarily, only two (types of) meanings: “a company producing smartphones,” based on the structure [[smart phone] company] and “a smart company producing phones,” based on the structure [smart [phone company]]. In natural language, meaning is not created by just “putting things together” such that “the whole assembly means something which is a reflection of the meanings of the parts” [12]. The example shows we have the same words and the same orders, and still we have different meanings dependent on different syntactic arrangements. Compositional meaning is ruled by hierarchical structure (syntax) and not by simply adding up individual meanings of the words combined. In other words, compounds in language are like compounds in chemistry, not mixtures. No chemist would even think of confusing compounds and mixtures.

Compositionality in human language involves a function that applies freely and productively, and these properties have not been shown to apply to the vocalizations of Japanese tits or southern pied babblers. Construing birdsong enrichments as “precursors” to human language compositionality is no more coherent than considering chemical mixtures as “ancestral states” to chemical compounds.

## How songbirds communicate meaning

**Fig 2:**
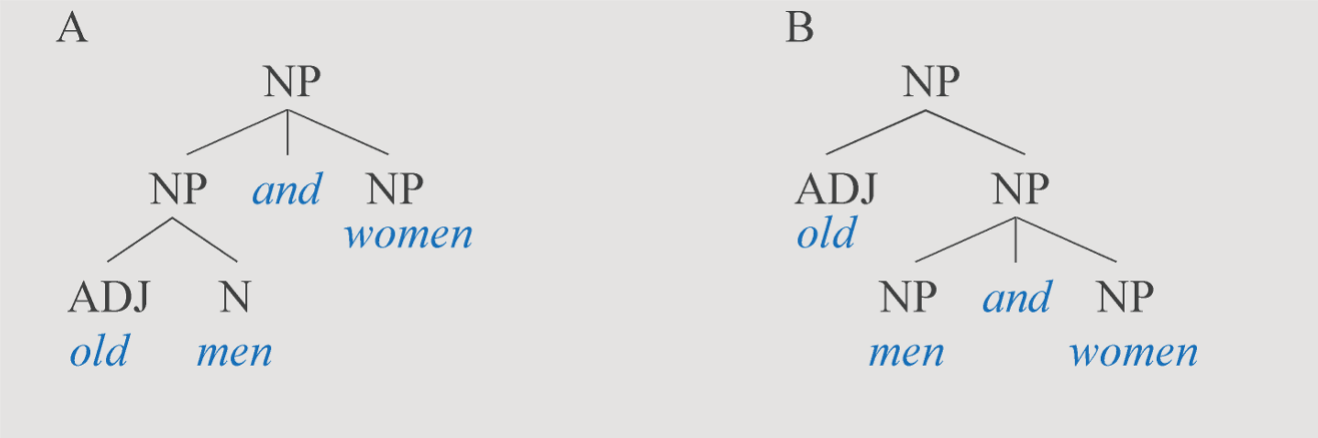
String arrangements. In Fig 2A the phrase is interpreted as [[old men] and [women]], i.e., “a collection of women (young or old) and old men.” In contrast, in Fig 2B, the phrase is interpreted as [old [men and women]], i.e., “a collection of men and women, all of them old.” ADJ, adjective; N, noun; NP, noun phrase

Suzuki and colleagues [3] describe compositional syntax in the context of bird calls as “whether receivers extract a compound meaning when both elements are combined.” For these authors, the fact that birds can combine an ABC call and a D call into an ABC-D call, this being a single meaningful unit, represents semantically compositional syntax. However, such a “compound meaning” does not necessarily make the meaning of a call compositionally derived from its constituent calls. Suppose we combine the word *old* with the words *men*, *and*, and *women*. In this case, it makes a large difference how these elements are (syntactically) combined. We can combine them as illustrated in Fig 2A or 2B, with different interpretations. In Fig 2A, the phrase is interpreted as [[old men] and [women]], i.e., “a collection of women (young or old) and old men.” In contrast, in Fig 2B, the phrase is interpreted as [old [men and women]], i.e., “a collection of men and women, all of them old.”

From the surface string *old-men-and-women*, one cannot immediately determine which is which, but their different arrangements in Fig 2A and 2B matter substantially to the interpretations we derive from the rules that are used to combine the constituent elements. Association of meaning with structure in natural language is essentially determined by the principle of “compositionality.” Given the difference in structure exemplified in Fig 2A and 2B, the meaning of the phrase will be different. In other words, the meaning of a complex unit is derived from its constituent parts, importantly, taking into account how these constituent parts are structurally combined. Crucially, natural language has hierarchical syntactic structure [9]. At the word and sentence level, serial ordering is not sufficient for building meaning (Box 2).

**Box 2: Order and meaning.**

To what extent does linear order play a role in human language? Less than one might be inclined to think. It seems straightforward that order affects meaning. Putting together the words *bird* and *song* will lead to either *birdsong* (“a type of song”) or *songbird* (“a type of bird”)—at the surface, a meaning difference depending on order. However, we have to realize that, in many instances, different orderings of elements need not lead to differences in meaning. The phrases *men and women* and *women and men* both mean the same thing, i.e., the set union of the classes of adult human males and adult human females. This is so because, from a compositionality perspective, these phrases are identical: the same words and the same syntactic arrangement.

There is a simple cross-linguistic analog to this language-internal illustration. The Japanese verb phrase *sakana o tabeta* (“ate a fish”) has the same meaning as the English verb phrase *ate a fish*. The composed elements are comparable (the noun phrase *a fish* approximates *sakana o*, and the finite verb *ate* approximates *tabeta*), and the rule by which these elements are combined to yield a verb phrase is the same. The only difference is the linear order of the elements combined: verb-object (English) versus object-verb (Japanese). But this is an ancillary property of language and forced only by the demands of the human sensorimotor system, which requires linear externalization. The particular order of the lexical elements varies arbitrarily between different languages. What is crucial here is that equivalent elements are combined by the same syntactic rule to yield identical determinate compositional meanings that are not freely negotiable. In other words, we get the right meaning through structure, not through order.

However, if we look at another level, the discourse level, things change. The sequence *how are you* // *I am feeling good* as an act of communicative behavior is sensitive to order. The sequence *I am feeling good* // *how are you* is “meaningless” from a communicative perspective. This is not a violation of compositionality, because this principle does not apply at the discourse level of language use.

## Building blocks

We still need to go one step further. Suzuki and colleagues [3,5], on the basis of earlier work [7], assert that calls have “meaning.” The suggestion is that calls, as units of meaning, are comparable to words. But what if calls, if at all comparable to linguistic meaning units, are to be compared with utterances? Note that both *fish* and *how are you*? are meaningful linguistic units. However, they have a different linguistic status. The former is a linguistic unit (word) that can be combined, through syntax, with other words into bigger chunks, phrases, and sentences. The latter, however, is a complete utterance, a conversational act (itself, of course, having a regular syntax) used as a polite greeting. It contributes to communicative behavior and combines with the answer *I am feeling good*, but the combination of the two—*how are you* // *I am feeling good*—falls beyond sentence grammar. It corresponds to the communicative use of language following Gricean principles of conversation, at which level syntax plays no role in the combination of elements. Such an example illustrates that one should be careful in distinguishing the study of communicative behavior—linguistic or nonlinguistic (like raising one’s hand as a greeting gesture)—from the study of meaning (semantics) in a narrower sense. Semantics studies how words and phrases can have a meaning that is independent of how they are used in communicative contexts. To give an example, *an interesting suggestion* means what it means, but it could, in a certain context, very well be used to convey that what is suggested is actually irrelevant.

Consequently, it is not at all clear that the songbird calls in these three studies are “compositional” in the conventional linguistic sense. The proposal in Suzuki and colleagues’ studies [3,5] that the ABC-D calls are “strung together”—the temporal ordering of responses correlating with the linear ordering of constituent calls—is a coherent position but only if the authors’ use of the term “composition” diverges from its use in natural language analysis. Suzuki and colleagues’ results refer to properties of “externalized communication” (vocalizations), sounds that are linearized, like “beads on a string.” As Suzuki and colleagues’ characterization of human language acknowledges, “recursive compositionality” is a property of natural language yielding an unbounded array of hierarchically structured phrases that are, in turn, linearized, or “flattened,” into a sequence of word-like atomic elements [9]. These experiments show no unbounded collection of compound calls nor hierarchically structured compound calls.

## “Linear compositionality?”

As we have discussed (Box 1), crucially, human language has a hierarchical syntactic structure. Furthermore, as we have seen (e.g., Fig 2), the meaning of a phrase is dependent on its syntactic structure. The authors of these three papers suggest that their findings indicate “potentially early forms of human syntactic communication” or “proto forms of compositional syntax” [4] or that the concept of compositionality can also be applied to serial order in a linear string [3]. In their most recent paper, Suzuki and colleagues [5] suggest that “compositionality depends on call ordering.” Although linear order sometimes does and sometimes does not play a role in human language syntax (as explained in Box 2), is it conceivable that these songbirds have a kind of “linear compositionality,” as the authors suggest?

The results of all three studies do not, in fact, support the suggestion of “linear compositionality,” in which meaning could be derived from call ordering. Engesser and colleagues [4] did not change the order of calls that were presented to their subjects. There was no difference between their experimental groups in the way in which the calls were “composed.” In their two studies, Suzuki and colleagues [3,5] did change the order of the two calls artificially. However, the birds did not respond to this novel order. The animals’ response would presumably have been the same if no call stimulus had been presented to them. It is not the case that a different serial order of the two calls had a different meaning. Rather, artificially placing the two calls in reverse order did not lead to a significant response in the birds—the novel compound call seemed to have no meaning.

It is important to point out that, apart from not leading to a response, the reversed call order in Suzuki and colleagues’ studies [3,5] was created artificially. In the natural situation, such combinations are not produced by the birds themselves. Further, the information provided in these three papers [3–5] does not suggest that the birds of either species productively vary the serial order of calls in the wild (even though variation has been observed [7]). In other words, these birds do not seem to employ their repertoire of calls in a generative way, producing different call combinations that have different meanings. In these studies, all the “composing” was done by the experimenters, while the birds do not appear to do that in the wild. Had it been the case that a different serial order of calls would have a different meaning, it is likely that the birds would have learned this early in life [5] rather than generating different combinations on the spot. This is in stark contrast to compositionality in human language, which not only involves hierarchical (rather than linear) structure [9] but is employed continually, productively, and creatively in human language use.

## Alternative explanations

We suggest that the behavioral performance of the birds in these studies [3–5] can be explained without the need for any syntactic operations shaping meaning. We argue that the differential responsiveness to the different combinations of calls constitutes a very interesting signaling system but involves no syntax and therefore no compositionality. Different calls can have different “meanings,” i.e., lead to different behavioral responses by the “receiver.” Thus, in the Japanese tits, the ABC call leads to an increase of scanning, and the D call leads to an increase in approach behavior, while ABC-D leads to both [3]. In other words, the compound of ABC and D leads to a different (compound) response than to either call by itself, which is an interesting observation. What about the lack of responsiveness to D-ABC, in which both the constituent elements (ABC and D) are also present? The authors state in their paper that D-ABC is “artificially reversed,” whereas ABC-D is a natural vocalization. But they do not appear to take this into account in their interpretation of the results. This is a very important issue because the lack of a response, or a reduced response, to a sound that is not a natural signal is not a priori unexpected in any animal communication system, and hence this outcome is not a solid basis for far-reaching conclusions on humanlike compositionality. In fact, a closer look at the results of both studies with Japanese tits [3,5] reveals that the birds show very little or no responsiveness to novel call combinations (Fig 4 in [3] and Figs 2 and 3 in [5]). The interesting result in the most recent of these studies [5] is that the birds do respond to the acoustically novel but “semantically” familiar combination of two familiar calls, a conspecific A followed by a heterospecific R (ABC-tää; see Fig 2 in [5]).

Furthermore, Suzuki and colleagues’ [3] reasoning is tacitly based on the assumption that perceptual meaning must directly lead to response behavior: if the D-ABC sequence would be interpreted by the birds as two consecutive calls “approach-scan,” then this should lead to corresponding increases in approaches and scans. Because this is not what the results show, the birds do not interpret it this way, which is seen as evidence for compositionality. Consider as an analogy the sentence *Jump off that bridge*. It has a clear meaning but will rarely lead to a corresponding response with this sequence of behaviors. To Japanese tit receivers, first scanning and then approaching may well be a more adaptive response order than the other way around. More generally, it is unlikely that upon predator detection, the sequence in which response behaviors occur is irrelevant. Receivers are not expected to carry out maladaptive response sequences, even if they were to interpret a signal as carrying such a meaning. As a consequence, it may be maladaptive for senders to produce such signals, which is why they are not found.

## Conclusions: The evolution of vocalization, speech, and language

These three studies on songbird calls have revealed very interesting and novel insights into vocal communication. Both avian species have a repertoire of calls that have meaning, i.e., that lead to a particular response when the birds are exposed to them. The babblers showed a greater response to a combination of an alert call and a recruitment call than to either call individually [4]. The Japanese tits displayed a “compound response” to the combination of their alert call and recruitment call [3]. Interestingly, these birds responded similarly to an artificially produced compound made up of a conspecific alert call and a heterospecific (but familiar) recruitment call [5]. Thus, the animals appeared to respond to a familiar combination of an alert call followed by a recruitment call, even though this combination was acoustically novel. This suggests that the animals responded to a combination of meaningful vocalizations rather than to a set combination of familiar sounds with a fixed meaning.

What are the implications of these interesting findings in songbirds [3–5] for the evolution of speech and language? As we have argued above, these studies do not conclusively establish the existence of compositionality in the vocalizations of these birds. To our knowledge, so far, there is no convincing evidence for this capacity in any nonhuman species [2,8,13]. The faculty of language may have evolved only once, in *Homo sapiens* [2,13,14]. It has been argued human language is likely to have emerged relatively recently in evolutionary time [13–15], possibly a few hundred thousand years ago; for a different view, see [16–18], among others. Auditory-vocal learning has evolved many times, in humans, songbirds, cetaceans, and other animals, but not in nonhuman primates—a case of convergent evolution [2,19]. To date, there is no evidence for convergent evolution of the capacity of hierarchical structure building of the type found in natural languages, and the results discussed here seem to further confirm this state of affairs.

## Abbreviations

A: alert call
AR: alert-recruitment call compound
C: chuck call
CR: compound of a chuck call and a recruitment call
R: recruitment call
